# Evaluation of Reaction Time during the One-Leg Balance Activity in Young Soccer Players: A Pilot Study

**DOI:** 10.3390/children10040743

**Published:** 2023-04-19

**Authors:** Fábio Saraiva Flôres, Joana Lourenço, Lucy Phan, Simon Jacobs, Renata Matheus Willig, Priscila Ellen Pinto Marconcin, Nuno Casanova, Denise Soares, Filipe Manuel Clemente, Ana Filipa Silva

**Affiliations:** 1 KinesioLab, Research Unit in Human Movement, Instituto Piaget, 1950-157 Lisboa, Portugal; 2 Research Center in Sports Performance, Recreation, Innovation and Technology (SPRINT), 4960-320 Melgaço, Portugal; 3 BlazePod, Play Coyotta Ltd., Tel Aviv 6971915, Israel; 4 Liberal Arts Department, American University of the Middle East, Egaila 15453, Kuwait; 5 Escola Superior Desporto e Lazer, Instituto Politécnico de Viana do Castelo, Rua Escola Industrial e Comercial de Nun’A’ lvares, 4900-347 Viana do Castelo, Portugal; 6 Instituto de Telecomunicações, Delegação da Covilhã, 1049-001 Lisboa, Portugal; 7 The Research Centre in Sports Sciences, Health Sciences and Human Development (CIDESD), 4800-058 Guimarães, Portugal

**Keywords:** soccer, motor behavior, motor development, performance, training, sports, simple reaction time, decision-making

## Abstract

This study’s aim was two-fold: (i) to test the intra-session reliability of the one-leg balance activity test; and (ii) to assess the influence of age on reaction time (RT) and the differences between dominant and non-dominant feet. Fifty young soccer players with an average age of 12.4 ± 1.8 years were divided into two groups: younger soccer players (n = 26; 11.6 ± 0.9 years) and older soccer players (n = 24; 14.2 ± 0.8 years). Each group then completed four trials (two with each leg) of the one-leg balance activity (OLBA) to evaluate RT under a single-leg stance. Mean RT and the number of hits were calculated, and the best trial was also selected. *T*-tests and Pearson correlations were performed for statistical analysis. Values for RT were lower, and the number of hits was higher while standing on the non-dominant foot (*p* = 0.01). MANOVA revealed that the “Dominant Leg” factor did not affect the multivariate composite (Pillai Trace = 0.05; F(4, 43) = 0.565; *p* = 0.689; Partial ETA Squared = 0.050; Observed Power = 0.174). The “Age” factor did not present an effect on the multivariate composite (Pillai Trace = 0.104; F(4, 43) = 1.243; *p* = 0.307; Partial ETA Squared = 0.104; Observed Power = 0.355). The results of the present investigation demonstrate that RT may be lower while standing on the non-dominant foot.

## 1. Introduction

To achieve sports success, athletes must develop and improve on a set of motor skills specifically related to the sport [1,2]. These skills have been extensively examined, with research studies consistently highlighting their importance for optimal performance [3,4]. However, motor skill acquisition is influenced by several variables, such as the environment, the actual skill, the individual’s characteristics, and age [2,5,6]. Furthermore, one of the most important tasks for young and experienced athletes is perceiving and integrating complex moving patterns while focusing on different task requirements [7,8]. Indeed, several studies have shown that attention and perception are essential to improving performance during soccer tasks, even in young athletes and practitioners [9,10,11].

Considering the vast perceptual and motor skills required to play soccer, reaction time (RT) has been highlighted as one of the essential physical indicators of performance [12,13], being frequently used as a measure to assess performance levels in many sports [4,14,15,16,17,18]. RT is commonly described as how long a person takes to prepare and initiate a movement, and therefore, it can be defined as the time interval between the onset of a not anticipated signal and the beginning of the movement [2,14,19]. Information processing, cognition, and RT are also seen as essential indicators of speed and effectiveness of decision-making in sports [20,21,22,23], which can greatly influence performance in soccer. Furthermore, research has demonstrated that RT tends to decrease from infancy into the late 20 s, contributing to an increase in performance [24,25], and it seems that RT is also associated with people’s attention capacity [26].

Specifically in soccer, disjunctive RT [27], choice RT [28,29], and simple RT [30] have been previously evaluated. However, most of these studies usually employ non-ecological methodologies instead of sport-related tasks and try to investigate experienced players instead of young soccer players [4,15,30,31,32,33,34].

Regarding soccer, not only is RT a critical indicator of performance, but it is also essential to recognize that athletes are frequently challenged under unstable conditions such as unipodal one-leg stances (e.g., decision-making during running or dribbling). Thus, balance is another critical variable for sports practitioners. The concept of balance refers to maintaining postural stability while stationary or moving (i.e., static, or dynamic balance, respectively), describing our capability to stand, sit, or move without falling [2,6]. The ability to sustain a balanced position plays a crucial role and might be considered an important indicator of performance in soccer [35,36]. As soccer frequently requires one-leg stances (e.g., kicking a ball while standing on one leg) [37], soccer players often demonstrate better static and dynamic balance abilities than other athletes whose sport mainly requires bipedal stances [16,35]. Considering the aforementioned evidence highlighting the importance of RT and balance for performance, it could be important to assess these variables in young soccer players.

Different methods have been developed and validated to assess RT and balance and are commonly used in sports research [33,38,39,40]. Light Sport Training System is a complex system commonly used for RT assessment, physical training, and coordination development [41,42,43]. Hence, this system can highlight perceiving and integrating complex moving patterns while allocating attentional resources in different critical areas of the dynamic scene. Despite the potential of this equipment, few studies have used this technology to assess RT under conditions in which balance is challenged [44]. Therefore, this study aimed to examine the RT of young soccer players during the “One-Leg Balance Activity” (OLBA). Specifically, this study aimed to assess the differences between dominant and non-dominant feet and the influence of age group on RT. Hence, we also aimed to test the intra-session reliability of OLBA. It was hypothesized that players would perform better when performing the task with the non-dominant foot on the floor and that younger soccer players would exhibit higher RT scores. Additionally, it was hypothesized that age, player experience, stature, body mass, and body mass index (BMI) would be associated with RT and hits.

## 2. Materials and Methods

### 2.1. Study Design and Setting

This study followed a cross-sectional study design. The assessment was performed between the 1st and 10th of June 2022 for the under-12 to under-16 teams and then, players were divided into two age groups: the younger soccer players (aged between 10 to 12.99 years) and older soccer players (aged between 13 to 15 years old). The period fits in the final part of the in-season, between the 40th and the 41st week after the start of the season. The study was conducted on a regular training day, before the training practice, and after 24 h of rest following the late training session or match. The assessments were performed in a controlled space during the evenings, in the university laboratory, with an environmental temperature of 20.4 degrees Celsius and relative humidity between 85% and 100% on all days. Participants were naïve regarding the task and could not see any demonstration or other players’ performance.

### 2.2. Participants

A convenience sampling strategy was performed. A total of 65 players were initially recruited in the same youth academy. Recruitment was performed by the investigators, and the order to experiment was randomly assigned. The eligibility criteria were: (a) participants needed to be soccer players for at least one season and participate in at least two training sessions per week; (b) players could not present any injuries, physical limitations, or diseases that could affect performance during the assessment’s tasks. From a total of 65 players recruited, 15 were excluded based on the following reasons: five had highly demanding physical activities on the day before the testing, six presented injuries and could not participate, two were not allowed by the legal guardians to participate, and two did not show up to training on the testing day. Participant characteristics can be observed in Table 1.

Oral and written consent was obtained from the participants (and their legal guardians) before the evaluation. This research was approved by the University Ethics Committee (ISEIT de Almada, Instituto Piaget, Portugal, P12-S21-21.06.22), and the study protocol followed the Declaration of Helsinki guidelines [45].

### 2.3. Procedures

Before the study began, methods were explained to the participants, and stature was recorded during inspiration using a stadiometer. All participants were asked to stand erect on the stadiometer with bare feet (SECA 213, *Bacelar & Irmão Lda*, Portugal). The horizontal bar of the stadiometer was placed on the vertex of the subject and the readings were recorded. To record body mass, a digital standing scale was used. The participant was asked to stand erect on the mechanical weighing machine with bare feet (SECA 761, *Bacelar & Irmão Lda*, Portugal). The readings were recorded from the scales of the digital weighing machine. Thus, the Body Mass Index (BMI) was calculated by: BMI = body mass (in kilograms)/stature^2^ (in meters) [46]. The same researcher with experience in anthropometric data collection performed the assessments. All participants were assessed in the evenings, with three hours since the last meal, and were using the regular training equipment, except the soccer boots.

All players were evaluated in a controlled environment (i.e., a quiet room in the university laboratory, without any external interference that could disrupt players’ attention) before their soccer training practice (6 PM), and all data collection was conducted under similar conditions. After the anthropometric assessments, participants performed a standardized warm-up protocol consisting of one-minute jogging and one-minute stretching. After the warm-up, participants were familiarized with the OLBA task. To familiarize themselves with the instrument and the specific task, participants performed two trials (one with each leg) before starting data collection. Between the familiarization trial and the first trial, the kicking leg was defined as the dominant leg, and the supporting leg as the non-dominant leg [47].

### 2.4. Task and Instrument

Light Sport Training System is a complex system comprised of wireless lights that light up randomly or in a pre-determined order and deactivate following the user’s touch. This system is used for RT assessment, physical training, and coordination development [41,42,43,44] to create visuomotor challenges via central and peripheral visual stimuli; thus, performance can be represented as either the number of completed responses (hits; as a balance measurement) or the average time between responses (milliseconds; as an RT measurement).

**Figure 1:**
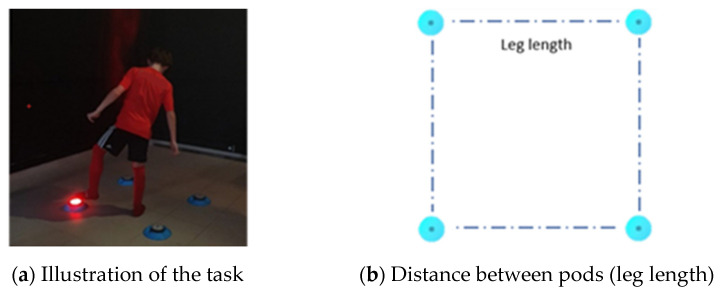
Representation of the OLBA.

The OLBA task (available on the BlazePod™, Tel Aviv/Israel) was used to examine RT on one-leg stance, a drill that assesses simple RT (milliseconds) and the total number of hits (i.e., touches on the pod). The OLBA task requires four pods arranged and placed on the floor in a square, with the player’s leg length (measured from the greater trochanter to the floor) as the distance between the pods (Figure 1). Participants positioned themselves at the center of the square while barefoot, balancing only on one foot for 30 s. The main goal of the activity is to tap on the highlighted pod (indicated by a light, which is lit in a rhythmic and random order) with the elevated foot as quickly as possible while maintaining balance on a one-leg stance. Participants could not touch the floor with the elevated foot more than one time in each trial. Participants that touched the floor with the elevated leg more than once had their test terminated and the results were excluded from the analysis. No feedback was provided to participants.

Two trials were made (after the one performed in the familiarization) with each leg (starting with two consecutive sets with the non-dominant feet followed by two with the dominant on the floor) interspersed with 30 s of resting between all sets. The total number of contacts with the pods (i.e., hits) was recorded as a balance performance and the RT of each trial as a decision-making performance. Thus, the best trial of each variable was used for data analysis.

The OLBA task requires an iPad or tablet to collect data. Thus, the researcher filters by the participant or by data, for example, and it is possible to download in an Excel^®^ sheet.

### 2.5. Statistical Analysis

Descriptive statistics with mean and standard deviation were calculated for the final sample to characterize the data. The Shapiro–Wilk test was used to examine the normality of distribution. All variables presented normal distribution and the Levene test confirmed the homogeneity of the sample, therefore, parametric tests were used. The sample size was divided into two groups, the younger athletes (aged between 10 to 12.99 years), and the older athletes (aged between 13 to 15 years old). The coefficient of variation (CV) was calculated considering each trial and all the trials together [48,49]. The intra-class coefficient correlation (ICC) was also determined among trials, considering a two-way fixed model [49]. ICC coefficients < 0.50 were considered poor, those between 0.50 and 0.75 were considered moderate, coefficients between 0.75 and 0.90 were considered good, and above 0.90 were considered excellent [48]. The paired sample *t*-test was used to analyze variations between trials (familiarization vs. 1st trial; familiarization vs. 2nd trial; and 1st trial vs. 2nd trial). The significance of the factors “Dominant leg” and “Age” on the variables “Maximum hits non-dominant leg”, “Maximum hits dominant leg”, “Maximum RT- non-dominant leg” and “Maximum RT-dominant leg”, was evaluated with a MANOVA after validating the assumptions of multivariate normality and homogeneity of variances-covariances. The assumption of homogeneity (Table 2) of variances-covariances in each group was evaluated with Box’s M test (M = 39.65; F (20, 488.18) = 1.43; *p* = 0.101). A significance level of a= 0.05 was considered. The Statistical Package for Social Sciences (SPSS; IBM Statistics, version 29.0) was used, adopting an alpha level of significance of 5%.

## 3. Results

The results are presented regarding balance and RT concerning players’ age groups. Table 3 compares trials for each leg and dependent variable (hits and RT). As it is showed in Table 3, for both the dominant and non-dominant legs, in balance and in RT, differences were observed between familiarization and trials (trial 1 and trial 2). However, between trials (trial 1 and 2), differences were not observed in the non-dominant leg for the older players for both balance and RT, and in the dominant leg in young players for both balance and RT.

Regarding the variability observed in the implemented tests, Table 4 presents the CV and the ICC between trials regarding hits and RT. Regarding trials 1 and 2, the results showed ICCs for balance ranged between 0.785 and 0.906, which indicates excellent reliability. Considering RT, ICCs ranged between 0.436 and 0.926, which indicates good (up to excellent) reliability.

**Figure 2:**
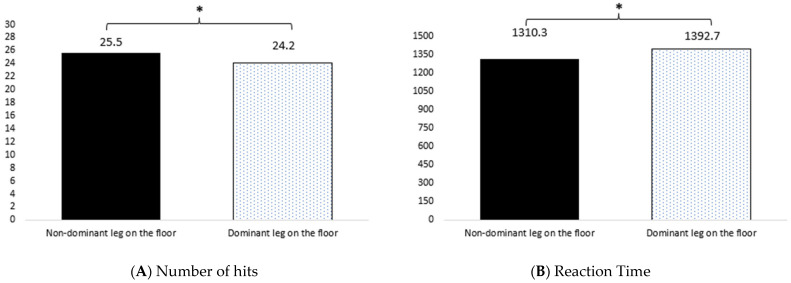
Comparisons between dominant and non-dominant leg. Note: * *p* < 0.05.

Figure 2 showed statistical differences between the non-dominant leg and the dominant leg regarding the hits (Figure 2A) best trial [t (49) = 2.609; *p* = 0.012] and the RT best trial [t (49) = −2.015; *p* = 0.049] (Figure 2B). When controlling the variables according to age group, the results showed no statistical significance to the hits best trial [t (26) = 1.877; *p* = 0.072] and RT best trial [t (26) = −1.142; *p* = 0.264] regarding the younger soccer players. The results also showed no statistical differences between the older soccer players regarding the hits best trial [t (24) = 1.78; *p* = 0.088] and showed statistically marginal differences regarding RT best trial [t (24) = −1.99; *p* = 0.058].

Table 5 shows the correlation between age, experience, stature, body mass, and BMI with the number of hits and RT (best trials) when controlling by age group. Results showed that age is associated with the number of hits and RT, especially in older players.

A multivariate analysis of variance (MANOVA) was performed to assess whether the factors under study had a statistically significant effect on the dependent variables (Table 6). MANOVA revealed that the “Dominant Leg” factor did not have an effect on the multivariate composite (Pillai Trace = 0.05; F(4, 43) = 0.565; *p* = 0.689; Partial ETA Squared = 0.050; Observed Power = 0.174). In a less conservative but more powerful tool for analysis, Roy’s Largest Root was used, obtaining the same conclusions regarding the absence of statistical significance. Regarding the “Age” factor, it also does not present an effect on the multivariate composite (Pillai Trace = 0.104; F(4, 43) = 1.243; *p* = 0.307; Partial ETA Squared = 0.104; Observed Power = 0.355). The same conclusions were obtained using Roy’s Largest Root. Finally, the interaction between factors did not have a statistically significant effect on the multivariate composite (Pillai Trace = 0.051; F(4.43) = 0.576; *p* = 0.683; Partial ETA Squared = 0.051; Observed Power = 0.176).

## 4. Discussion

The present investigation examined the RT of young soccer players on a one-leg stance during the OLBA task. It was hypothesized that performance would be different regarding foot dominance, and that older soccer players would exhibit lower RT values and a higher number of hits compared to younger soccer players. Age, player experience, stature, body mass, and BMI were also expected to be associated with RT and the number of hits. The present manuscript’s main findings do not agree with our hypotheses. RT and the number of hits does not show any statistical differences while players had the non-dominant foot on the floor. These results disagree with other investigations that showed the benefits of sports and exercise in reducing RT and improving balance [12,14,50]. Several studies have reported associations between physical activities with RT [12,14,15,51,52] and balance in different populations [50,51,53]. For example, Misra et al. [54] found that the right foot outperformed the left during a visual RT task performed by young adults. However, the authors did not mention foot preference. Like our results, Bigoni et al. [35] found no differences between dominant and non-dominant single-leg stance control among young soccer players. However, the authors did not use an ecological task to assess balance scores.

Much research also reported that perceiving and integrating complex moving patterns while focusing on different task requirements is key to better performance levels [7,8,11,55]. Pinheiro et al. [13] compared the discriminative RT in elite young soccer players (U-15 and U-17) using the Vienna Test System SPORTS^®^ to assess RT. Results showed significant differences between categories, with the older players outperforming their younger counterparts. Using a similar age to our investigation, Rodrigues et al. [32] evaluated balance levels and RT of primary school children who have or have not ever been overseen by a physical education teacher. Their results showed that children with physical education teachers presented better levels of balance and outperformed children without physical education teachers regarding RT.

Literature has also investigated the relationship between soccer player position and RT. Ruschel et al. [56] analyzed soccer players’ visual and auditory simple RT from differing categories and field positions. Despite not being the same task as our investigation, which exclusively uses visual RT, Ruschel and colleagues found that goalkeepers showed significantly faster visual RT than midfielders. One reason pointed out by the authors was that players from differing categories and field positions may adopt more complex strategies in retaining and using visual information in specific situations. Finally, since soccer is essentially a visual game, visual RT may be more important to develop than auditory RT.

Concerning balance, Ricotti et al. [4] analyzed static and dynamic balance, visual RT, and acoustic RT of soccer players, observing that higher static balance performances in comparison with non-professional ones characterized high-level athletes. However, like our results, their findings regarding RT were not conclusive. Concerning the relationship between RT and balance, the authors mentioned above stated that there was no difference between gymnasts and non-gymnasts in balance and RT measurement [57], and similar findings were found with elite man badminton players [58]. Similarly, Drews et al. [15] assessed simple choice RT levels in young futsal and volleyball players, observing no differences between the two sports. Nevertheless, the authors did not include a control group to understand the real effect of sports participation on RT. An important consideration is that these perceptual-cognitive abilities, critical for elite performance, may be trainable [59]. As hypothesized by Cross and colleagues, sensorimotor brain regions may be engaged during action perception since such activation may enable athletes to predict the ongoing actions of others. Therefore, if these experiences are continuously trained, the athletes’ ability to predict and respond to uncertain in-game scenarios may be improved, which could significantly improve elite performance.

### Limitations and Future Directions

Previous investigations, especially regarding RT, failed to present an ecological task to assess RT [15,32,51,60]. For instance, most previous investigations used simple software computer tasks and not a real-life scenario (i.e., lower ecological validity) such as the one utilized in the present study. This is of great importance, as RT may be context-specific; thus, a lower RT during a computer task may not directly transfer to a lower RT and improved performance in sports. Therefore, we believe that the OLBA task can be an important tool that could be employed in future investigations, permitting a standardized examination of balance and RT in different populations. Nonetheless, the present study showed some pitfalls. First, this study assessed simple RT, but not choice RT, which is a critical component for soccer players considering the importance of decision-making in this sport [31]. Furthermore, due to the recency of this equipment and specific task, the absence of specific guidelines (e.g., test termination criteria, shoe type, or hand position) made it harder to standardize between participants. It should be noted that the low power of the test, combined with the small sample size, may be responsible for the lack of statistical significance.

Therefore, future studies should explore the validity and reliability of this task. Additionally, it could be important that new investigations aim to replicate these findings and use variations of this task (in standardized conditions) to assess not only simple RT but also choice RT and discriminatory RT. Hence, future studies should include a control group or a group of individuals who are mostly sedentary or not performing sports. This would allow the examination of whether sports participation is associated with lower RT, as has been postulated.

## 5. Conclusions

The results of the present investigation demonstrate that RT may be lower while standing on the non-dominant foot. Considering the potential trainability of these skills, it could be postulated that using ecologically tools could have a critical role in improving longer-term elite performance levels. Future studies should aim to use this technology as it has the potential to improve scientific knowledge and move this research field forward regarding RT assessment (and potentially improvement) in different populations, contexts, and sports.

## Figures and Tables

**Table 1:** Players’ demographic information.

	Younger Soccer Players	Older Soccer Players
N	26	24
Age (y)	11.65 ± 0.9	14.27 ± 0.8
Stature (meters)	1.46 ± 0.1	1.65 ± 0.1
Body mass (Kg)	37.60 ± 8.9	53.83 ± 8.7
Body mass index (Kg/m^2^)	17.32 ± 1.9	19.62 ± 2.6

**Table 2:** Box’s Test of Equality of Covariance Matrices.

Box’s M	39.653
F	1.433
df1	20
df2	488.184
Sig.	0.101

**Table 3:** Mean and standard deviation of the OLBA task.

Leg Preference	Age Group	Tests	Familiarization		Trial 1		Trial 2		Fam vs. Trial 1		Fam vs. Trial 2		Trial 1 vs. Trial 2	
			Mean	Sd	Mean	Sd	Mean	Sd	t	*p*	t	*p*	t	*p*
Non-dominant leg	Younger players	Balance	16.92	5.66	22.04	4.76	23.88	4.31	−6.76	0.001 *	−7.59	0.001 *	−2.64	0.014 *
		RT	2026.52	1687.73	1370.96	354.40	1244.04	235.14	2.27	0.034 *	2.33	0.031 *	2.08	0.048 *
	Older players	Balance	18.96	6.047	25.50	4.98	25.37	5.60	−4.067	0.001 *	−4.34	0.001 *	−0.64	0.53
		RT	1488.90	484.57	1160.71	269.47	1138.42	240.49	2.22	0.038 *	2.59	0.018 *	1.24	0.23
Dominant leg	Younger players	Balance	18.12	4.84	21.50	5.57	21.92	4.816	−7.89	0.001 *	−5.40	0.001 *	0.14	0.89
		RT	1944.24	111.92	1418.69	378.59	1354.69	268.13	4.56	0.001 *	4.12	0.001 *	0.81	0.43
	Older players	Balance	19.83	5.35	23.38	5.21	25.00	5.18	−3.043	0.006 *	−4.61	0.001 *	−2.93	0.008 *
		RT	1550.67	425.80	1274.54	293.26	1177.58	241.41	3.20	0.004 *	4.45	0.001 *	3.49	0.002 *

Note: * *p* < 0.05; balance (number of hits); RT—reaction time (measured in milliseconds); Fam—familiarization; Sd: standard deviation.

**Table 4:** Coefficient of variation (CV) and intra-class coefficient correlation (ICC) between trials.

Leg Preference	Age Group	Tests	Between Familiarization and Trial 1		Between Familiarization and Trial 2		Between Trial 1 and Trial 2	
			CV	ICC	CV	ICC	CV	ICC
Non-dominant leg	Younger players	Balance (hits)	21.76	0.66	26.92	0.45	10.04	0.79
		Reaction Time (ms)	19.07	0.50	21.14	0.17	7.42	0.44
	Older players	Balance (hits)	23.60	0.61	25.30	0.48	5.60	0.80
		Reaction Time (ms)	20.53	0.60	25.81	0.49	9.19	0.930
Dominant leg	Younger players	Balance (hits)	17.23	0.78	18.20	0.61	10.01	0.88
		Reaction Time (ms)	22.50	0.20	23.15	0.12	9.75	0.80
	Older players	Balance (hits)	17.42	0.52	19.76	0.480	6.26	0.91
		Reaction Time (ms)	18.98	0.30	22.49	0.26	7.23	0.89

Note: ms—milliseconds.

**Table 5:** Correlations between age, experience, stature, body mass, and BMI with the number of hits and RT controlled by age group.

Age Group	Variables	Hits Best Trial		Reaction Time Best Trial	
		Non-Dominant Leg	Dominant Leg	Non-Dominant Leg	Dominant Leg
Younger players	Age (y)	0.37 *	0.17	−0.10	−0.02
	Experience (y)	0.24	0.23	−0.25	−0.33
	Stature (meters)	0.09	−0.17	0.15	0.13
	Body mass(Kg)	−0.10	−0.29	0.32	0.22
	Body mass index (Kg/m^2^)	−0.28	−0.35	0.45 *	0.26
Older players	Age (y)	0.52 *	0.44 *	−0.53 *	−0.43 *
	Experience (y)	−0.08	−0.30	0.08	0.20
	Stature (meters)	0.21	0.24	−0.09	−0.25
	Body mass(Kg)	0.19	0.29	−0.07	−0.24
	Body mass index (Kg/m^2^)	0.10	0.18	−0.03	−0.15

Note: * *p* < 0.05.

**Table 6:** Multivariate Tests.

Effect		Value	F	Hypothesis df	Error df	Sig.	Partial Eta Squared	Noncent. Parameter	Observed Power ^c^
Intercept	Pillai’s Trace	0.99	3308.26 ^b^	4.000	43.000	<0.00	0.99	13,233.04	1.00
	Wilks’ Lambda	0.00	3308.26 ^b^	4.000	43.000	<0.00	0.99	13,233.04	1.00
	Hotelling’s Trace	307.75	3308.26 ^b^	4.000	43.000	<0.00	0.99	13,233.04	1.00
	Roy’s Largest Root	307.75	3308.26 ^b^	4.000	43.000	<0.00	0.99	13,233.04	1.00
Dominant Leg	Pillai’s Trace	0.05	0.57 ^b^	4.000	43.000	0.69	0.05	2.26	0.17
	Wilks’ Lambda	0.95	0.57 ^b^	4.000	43.000	0.69	0.05	2.26	0.17
	Hotelling’s Trace	0.05	0.57 ^b^	4.000	43.000	0.69	0.05	2.26	0.17
	Roy’s Largest Root	0.05	0.57 ^b^	4.000	43.000	0.69	0.05	2.26	0.17
Age	Pillai’s Trace	0.10	1.24 ^b^	4.000	43.000	0.31	0.10	4.97	0.35
	Wilks’ Lambda	0.90	1.24 ^b^	4.000	43.000	0.31	0.10	4.97	0.35
	Hotelling’s Trace	0.12	1.24 ^b^	4.000	43.000	0.31	0.10	4.97	0.35
	Roy’s Largest Root	0.12	1.24 ^b^	4.000	43.000	0.31	0.10	4.97	0.35
Dominant Leg * Age	Pillai’s Trace	0.051	0.58 ^b^	4.000	43.000	0.68	0.051	2.30	0.18
	Wilks’ Lambda	0.94	0.58 ^b^	4.000	43.000	0.68	0.051	2.30	0.18
	Hotelling’s Trace	0.054	0.58 ^b^	4.000	43.000	0.68	0.051	2.30	0.18
	Roy’s Largest Root	0.054	0.58 ^b^	4.000	43.000	0.68	0.051	2.30	0.18

Design: Intercept + Mem_Dom + Ag_grups + Mem_Dom * Age_grups ^b^ Exact statistic ^c^ Computed using alpha = 0.05.

## Data Availability

No new data were created or analyzed in this study. Data sharing is not applicable to this article.
